# Different initiatives across Europe to enhance losartan utilization post generics: impact and implications

**DOI:** 10.3389/fphar.2014.00219

**Published:** 2014-10-08

**Authors:** James C. Moon, Brian Godman, Max Petzold, Samantha Alvarez-Madrazo, Kathleen Bennett, Iain Bishop, Anna Bucsics, Ulrik Hesse, Andrew Martin, Steven Simoens, Corinne Zara, Rickard E. Malmström

**Affiliations:** ^1^Heart Hospital Imaging Centre, The Heart Hospital, University College HospitalLondon, UK; ^2^Division of Clinical Pharmacology, Department of Laboratory Medicine, Karolinska Institutet, Karolinska University Hospital HuddingeStockholm, Sweden; ^3^Medicine Use and Health, Strathclyde Institute of Pharmacy and Biomedical Sciences, University of StrathclydeGlasgow, UK; ^4^National Institute for Science and Technology on Innovation on Neglected Diseases, Centre for Technological Development in Health, Oswaldo Cruz Foundation (Fiocruz)Rio de Janeiro, Brazil; ^5^Occupational and Environmental Medicine, Centre for Applied Biostatistics, University of GothenburgGothenburg, Sweden; ^6^Department of Pharmacology and Therapeutics, Trinity Centre for Health Sciences, St. James HospitalDublin, Ireland; ^7^Public Health and Intelligence Business Unit, NHS National Services ScotlandEdinburgh, UK; ^8^Department of Finance, Faculty of Business, Economics and Statistics, University of ViennaVienna, Austria; ^9^Hauptverband der Österreichischen SozialversicherungsträgerVienna, Austria; ^10^National Institute for Health Data and Disease ControlCopenhagen, Denmark; ^11^NHS Greater Manchester Commissioning Support UnitSalford, Manchester, UK; ^12^KU Leuven Department of Pharmaceutical and Pharmacological SciencesLeuven, Belgium; ^13^Barcelona Health Region, Catalan Health ServiceBarcelona, Spain; ^14^Clinical Pharmacology Unit, Department of Medicine, Karolinska Institutet, Karolinska University Hospital SolnaStockholm, Sweden

**Keywords:** losartan, generics, demand-side measures, cross-national study, drug utilisation, Europe

## Abstract

**Introduction**: There is an urgent need for health authorities across Europe to fully realize potential savings from increased use of generics to sustain their healthcare systems. A variety of strategies were used across Europe following the availability of generic losartan, the first angiotensin receptor blocker (ARB) to be approved and marketed, to enhance its prescribing vs. single-sourced drugs in the class. Demand-side strategies ranged from 100% co-payment for single-sourced ARBs in Denmark to no specific measures. We hypothesized this heterogeneity of approaches would provide opportunities to explore prescribing in a class following patent expiry.

**Objective**: Contrast the impact of the different approaches among European countries and regions to the availability of generic losartan to provide future guidance.

**Methodology**: Retrospective segmented regression analyses applying linear random coefficient models with country specific intercepts and slopes were used to assess the impact of the various initiatives across Europe following the availability of generic losartan. Utilization measured in defined daily doses (DDDs). Price reductions for generic losartan were also measured.

**Results**: Utilization of losartan was over 90% of all ARBs in Denmark by the study end. Multiple measures in Sweden and one English primary care group also appreciably enhanced losartan utilization. Losartan utilization actually fell in some countries with no specific demand-side measures. Considerable differences were seen in the prices of generic losartan.

**Conclusion**: Delisting single-sourced ARBs produced the greatest increase in losartan utilization. Overall, multiple demand-side measures are needed to change physician prescribing habits to fully realize savings from generics. There is no apparent “spill over” effect from one class to another to influence future prescribing patterns even if these are closely related.

## Introduction

Health authorities across Europe could realize considerable savings through greater use of generic medicines. Between 2008 and 2013, the global annual sales of medicines losing their exclusivity was US$50 to 100 billion (€35–70 billion), reaching US$255 billion by 2016 (Frank, 2007; Jack, 2008; Godman et al., 2014). Once one or more drugs in a class lose their patent, and all drugs in the class are seen as essentially therapeutically similar at appropriate doses, it is legitimate for health authorities to encourage physicians to preferentially prescribe low cost generics to realize considerable savings without compromising care (Voncina et al., 2011; Godman et al., 2014). However, physicians and authorities do not always take full advantage of the availability of generics (Godman et al., 2010a, 2014). The availability of generic losartan provides an exemplar case to review health authority activities across Europe and the subsequent implications.

Renin-angiotensin inhibitor drugs had global sales of US$27.3 billion in 2010, 24% in Europe, much of this for single-sourced angiotensin receptor blockers (ARBs) (IMS Institute for Health Informatics, 2011). Broadly speaking, all angiotensin converting enzyme inhibitors (ACEIs) and ARBs are seen as having similar effectiveness for the management of hypertension and heart failure at appropriate dose titration (Heran et al., 2008; Moon et al., 2010; Voncina et al., 2011; Bucsics et al., 2012; Svanstrom et al., 2012; Godman et al., 2013a; Simoens et al., 2013). Patients in the UK have also been successfully switched between different ARBs without compromising care (Usher-Smith et al., 2008; Moon et al., 2010). However, other authors disagree believing losartan is inferior to other ARBs (Makani et al., 2014).

A variety of strategies were instigated by national and regional health authorities across Europe to preferentially encourage the prescribing of losartan once generic losartan became available. These included 100% co-payment for single-sourced ARBs in Denmark unless there was a good medical rationale, which effectively de-listed single-sourced ARBs (Hesse et al., 2013). Other strategies including removing prescribing restrictions for losartan but not for single-sourced ARBs, instigating prescribing targets for losartan vs. single-sourced ARBs, physician financial incentive schemes and therapeutic switching programmes (Bucsics et al., 2012; Godman et al., 2013a; Simoens et al., 2013; Martin et al., 2014). However, some authorities did nothing (Bennie et al., 2013; Godman et al., 2013b). We hypothesized this heterogeneity of approaches offers the opportunity for a “natural experiment” of prescribing in a class following patent expiry, and to benchmark the effectiveness and financial utility of different interventions to provide insight for future opportunities.

Consequently, the aim of the study is to compare and contrast the impact of the many different demand-side approaches among European countries following the availability of generic losartan. Only Western European countries and regions were chosen as typically losartan was available as a generic much earlier among Central and Eastern European countries; alternatively only generic losartan was reimbursed and not single-sourced ARBs (Voncina et al., 2011; Kalaba et al., 2012; Markovic-Pekovic et al., 2012). The objective being to provide guidance on potential future approaches that the authorities could consider as they seek to introduce additional measures to further enhance their prescribing efficiency.

## Methodology

The utilization of the different ARBs was calculated in terms of defined daily doses (DDDs), with 2011 DDDs used in line with International guidance (Ronning et al., 2000; Vlahovic-Palcevski et al., 2010). DDDs are recognized as the international standard to assess utilization patterns within and between countries (WHO, 2003; Bennie et al., 2013). Only administrative databases were used to assess utilization patterns. Details of these are included in Box 1.

Box 1 **Details of administrative databases use in the study and the date when generic losartan first reimbursed/included in the drug tariff (Coma et al., 2009; Bucsics et al., 2012; Cahir et al., 2012; Bennie et al., 2013; Godman et al., 2013a; Hesse et al., 2013; Simoens et al., 2013).**

- **Austria:** Internal data warehouse of the HVB (Hauptverband der Österreichischen Sozialversicherungsträger)—BIG—coupled with Cube HMSTAT, based on the “maschinelle Heilmittelabrechnung,” which covers approximately 98% of the Austrian population. Generic losartan was reimbursed from October 2008.
- **Belgium:** Pharmanet—a database of reimbursed medicines dispensed in ambulatory care in Belgium. This database is maintained by the National Institute for Health and Disability Insurance and covers the whole Belgian population. Generic losartan was reimbursed from July 2010.
- **Denmark:** Danish Prescription Registry covering the entire Danish population. Generic losartan was reimbursed from April 2010.
- **Ireland:** The National Shared Services Primary Care Reimbursement Service of the Health Service Executive in Ireland (HSE-PCRS) pharmacy claims database. This database provides details on monthly dispensed medications for each individual within the General Medical Services (GMS) population. The GMS population covers approximately 30% of the population of Ireland with higher morbidity than the general population—reflected in consuming approximately 65% of total pharmaceutical expenditure in Ireland. Generic losartan was reimbursed in Ireland from March 2010.
- **Scotland:** NHS National Services Scotland Corporate Warehouse covering the entire population in Scotland. Generic losartan was reimbursed (in the Drug Tariff) from July 2010.
- **Spain (Catalonia)**: DMART (Catalan Health Service) database covering all patients in the public system in Catalonia. Generic losartan was reimbursed from July 2006.
- **Sweden:** National Swedish Pharmacy Register covering the entire Swedish population. Generic losartan was reimbursed from March 2010.

Losartan utilization was subsequently converted into a percentage of total ARB utilization (DDD basis) before and after the availability of generic losartan (time zero) to enable meaningful comparisons between the various European countries factoring in differences in population sizes, time when generic losartan became available (Table 1) and the database characteristics between the countries (Box 1). The xtmixed command in Stata versions 12/13 (StataCorp, College Station, Texas, USA) was used to fit a linear random coefficient model with country specific intercepts and slopes. The model included a change in the slope at the time of introducing generic losartan in each country. Data on the number of monthly reimbursed prescriptions for all patients within each country's health service prescribed a minimum of one ARB (C09CA01 to 09) (Bucsics et al., 2012; Godman et al., 2013a; Simoens et al., 2013; WHO Collaborating Centre for Drug Statistics Methodology, 2013) at least 7 months before the availability of generic losartan in each country until at least 13 months after was used in the analysis.

**Table 1:** **Details of specific policies initiated among European countries following the availability of generic losartan**.

**Country**	**Start of reimbursement for generic losartan**	**Activities**
Austria	October 2008	Economics and Enforcement: Prescribing restrictions removed for losartan but not the other ARBs—ambulatory care physicians still required to document the rationale for prescribing a patented (single-sourced) ARB vs. an ACEI. The documents are subject to review by the health insurers Potential sanctions for abuse include physicians paying back to the Austrian Health Insurers an estimate of the increased drug expenditure if any abuse of the reimbursement restrictions is subsequently proven
Belgium	July 2010	Economics and Enforcement Status of losartan changed from a “chapter IV” medicine to a “chapter I” medicine; however, patented (single-sourced) ARBs remained chapter IV A chapter IV medicine can only be prescribed subject to prior approval—otherwise a 100% co-payment. A chapter I medicine can be prescribed without restrictions
Denmark	April 2010	Enforcement Delisting of all other ARBs than losartan from the reimbursed list Patients could still be prescribed another ARB and have this reimbursed. However, the prescribing physician has to justify the rationale to the authorities and have the explanation accepted before other ARBs can be reimbursed. Otherwise patients are subject to 100% co-payment
Ireland	March 2010	No specific activities were undertaken to influence the prescribing of losartan vs. single-sourced ARBs
Scotland	July 2010 (Drug Tariff)	No specific activities were undertaken to encourage the preferential prescribing of losartan vs. patented (single-sourced) ARBs in view of other identified priorities by NHS Scotland as well as the imminent launch of generics of other ARBs including candesartan, irbesartan, and valsartan
		Ongoing multiple measures generally (Education, engineering, and economics) to encourage the prescribing of generic ACEIs vs. ARBs
Spain (Catalonia)	July 2006	No specific activities encouraging the preferential prescribing of losartan apart from highlighting standard costs/DDD for ACEIs and ARBs in physician contracts
Sweden	March 2010	Education, engineering, economics, and enforcement Education—County (Region) Drug and Therapeutics Committees encouraging the prescribing of generic losartan; changes in county prescribing guidance, guidelines, and formularies to recommend losartan first line for the management of hypertension or heart failure when an ARB is indicated; academic detailing endorsing losartan as the ARB of choice; monitoring prescribing habits against agreed guidance and feeding back the findings Engineering—Prescribing targets, e.g., % losartan as a % of all ARBs (DDD based); therapeutic switching programmes were also initiated by some Counties (regions) to change patients on single-sourced ARBs to losartan Economics—Budget devolution combined with positive or negative financial rewards to physicians to encourage them to stay within budget; revision of physician or practice based financial incentives to now include the prescribing of losartan vs. single-sourced ARBs Enforcement—From May 2011, prescribing restrictions were lifted for losartan but not the other ARBs. In addition, originator losartan was removed from the reimbursement list

Separate analyses were undertaken in Austria, Belgium, Denmark, Scotland and Sweden to assess whether the changes in losartan utilization patterns post generics were significant or not (Bucsics et al., 2012; Bennie et al., 2013; Godman et al., 2013a; Hesse et al., 2013; Simoens et al., 2013). These were retrospective segmented regression analyses of an interrupted time series design following the availability of generic losartan (Wagner et al., 2002). In three of the countries, serial autocorrelations of losartan prescription items dispensed were assessed with an ARIMA model using a Box-Jenkins-Tiao strategy (McDowall et al., 1980; Bennie et al., 2013; Godman et al., 2013a; Simoens et al., 2013), with common segmented regression models used to fit a least-squares regression line to each segment (Ross-Degnan et al., 1993). The Durbin-Watson statistic was calculated in each country to test for a serial autocorrelation of the error terms in the regression models to see whether significance was reached (*P* < 0.05) (Durbin and Watson, 1951; Brennan and Croft, 1994). In Austria, regression analyses were undertaken using the “R Development Core Team” methodology to ascertain whether the change in losartan utilization post generics was significant (Bucsics et al., 2012; R Development Core Team, 2012). In the linear regression analysis in Denmark, dummy variables were added to allow a change in intercept and in slope in April 2010, when generic losartan was reimbursed, to test for significance (Wagner et al., 2002; Hesse et al., 2013). Further details can be found in the country specific publications.

A separate analysis was also undertaken in one English primary care organization, NHS Bury, using a similar methodology to Belgium, Scotland and Sweden (Martin et al., 2014). However in this case, the unit of measurement was prescription items dispensed rather than DDDs as this is the typical metric used to assess and compare utilization patterns in England between different primary care groups (Martin et al., 2014). The rationale for including this regional primary care organization was that initially there were no specific demand-side measures introduced following the availability of generic losartan; however, multifaceted measures were introduced 7 months later including therapeutic switching to help realize considerable savings (Martin et al., 2014). Consequently this provides an additional exemplar case history of a regional health authority changing its policies over time.

The percentage of losartan dispensed as generics vs. the originator was also calculated in all the European countries and regions studied apart from Belgium to ascertain whether there were any problems with generic losartan in clinical practice. Fixed dose ARB combinations (FDCs) were not included in this research as FDC utilization can be as low as 2% of total renin-angiotensin inhibitor drug utilization in some countries (Voncina et al., 2011; Bennie et al., 2013). There is also continuing controversy surrounding the patient benefits of FDCs vs. titrating single agents separately, especially if FDCs have higher drug acquisition costs than the combination of each agent separately (Regional Drugs and Therapeutics Centre (NHS), 2008; Voncina et al., 2011; Kalaba et al., 2012).

We also calculated the percentage reduction in reimbursed expenditure/ DDD for generic losartan vs. pre-patent loss originator prices over time as well as the influence of generic losartan on subsequent ARB expenditure in separate analyses in a number of countries. As a result, compare the influence of the different policies on overall ARB prescribing efficiency with all ARBs seen as essentially similar at appropriate dose titration (Heran et al., 2008; Moon et al., 2010; Svanstrom et al., 2012).

The European countries and regions chosen for the study provided a range of geographical locations, different population sizes, different approaches to the financing of health care, i.e., taxation or health insurance based, as well as different approaches to enhancing the utilization of generics vs. originators and their pricing (Godman et al., 2009, 2014; Abuelkhair et al., 2012; Simoens, 2012; Vogler, 2012). Policies to encourage the prescribing of generics vs. originators included prescribing targets, e.g., Belgium, financial incentives either for physicians, patients, or both, e.g., Austria, Belgium and Spain, encouraging voluntary International non-proprietary name (INN) prescribing, e.g., UK, and compulsory generic substitution, e.g., Sweden (Godman et al., 2009, 2013c; Simoens, 2012; Simoens et al., 2013). Pricing policies for generics included prescriptive pricing policies as well as market forces encouraging their prescribing and dispensing (Godman et al., 2012a, 2013c; Vogler, 2012). This methodology is in line with recommended guidance for undertaking cross national comparisons (Cacace et al., 2013).

The demand-side measures were collated under the 4Es—Education, engineering, economics, and enforcement (Wettermark et al., 2009). These include (Godman et al., 2009, 2014; Wettermark et al., 2009, 2010; Garuoliene et al., 2011; Gustafsson et al., 2011; Voncina et al., 2011; Markovic-Pekovic et al., 2012):

- **Education:** Activities range from printed guidelines to more intensive strategies including academic detailing and benchmarking physician prescribing habits.
- **Engineering:** Refers to organizational or managerial interventions, e.g., prescribing targets and therapeutic switching initiatives.
- **Economics:** includes financial incentives for physicians, patients or pharmacists.
- **Enforcement:** includes regulations by law such as prescribing restrictions for ARBs in Austria, the Republic of Srpska and Sweden as well as compulsory generic substitution or compulsory INN prescribing.

A narrative review of health policies was undertaken in each country and region following the availability of generic losartan by one of the co-authors (Brian Godman). This was subsequently checked with each co-author, in line with previous cross-national studies (Godman et al., 2010a; Voncina et al., 2011).

## Results

The various European countries and regions approached the opportunity of generic losartan differently (Table 1). These policies were in addition to general policies to enhance the prescribing of generics which, as mentioned, included financial incentives for physicians and patients, prescribing targets, compulsory generic substitution and high voluntary INN prescribing (Godman et al., 2008, 2009; Coma et al., 2009; Bennie et al., 2013; Simoens et al., 2013).

**Figure 1:**
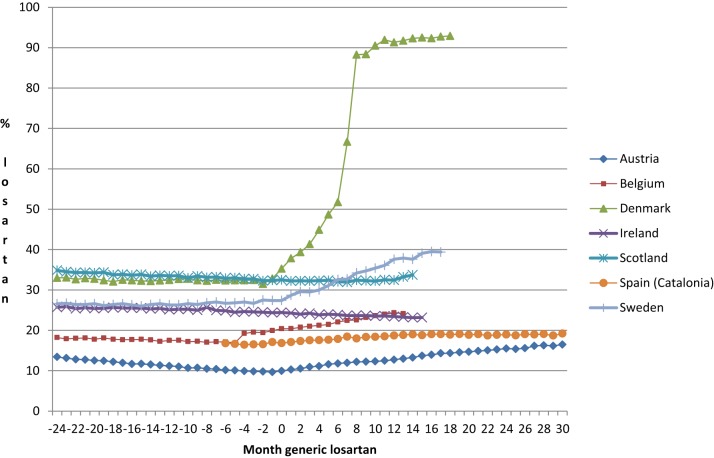
**Percentage utilization of losartan vs. all single ARBs (DDD basis) before and after the availability of generic losartan (Time 0) on a monthly basis**.

This resulted in substantial differences in the subsequent utilization of losartan vs. other ARBs (Figure 1). By changing the reimbursement status of patented (single-sourced) ARBs, e.g., Denmark, the utilization of losartan rose to over 90% of all ARBs (DDD basis) by the end of the study period.

There was an average increase in the utilization of losartan of 0.82% units per month among the various European countries and regions after the introduction of generic losartan (month 0), with a large variability among them (Figure 1). The standard deviation (SD) was 1.33% per month (Table 2).

**Table 2:** **Average change in regression slopes after the introduction of generic losartan and corresponding standard deviations over different groupings of the included countries**.

**Countries**	**Change in slope % units per month (95% CI)**	**Standard deviation of the change in slope *SD* (95% CI)**
All	0.82 (−0.17 to 1.82)	1.33 (0.78 to 2.26)
Excluding Denmark	0.30 (0.04 to 0.56)	0.32 (0.18 to 0.57)
Excluding Denmark and Sweden	0.22 (0.02 to 0.43)	0.23 (0.12 to 0.43)
Excluding Denmark, Sweden, Austria, Belgium	0.10 (0.01 to 0.20)	0.08 (0.03 to 0.19)

Separate country analyses showed a significant difference in the utilization of losartan post generics in Austria, Belgium, Denmark, and Sweden but not Scotland (further details can be found in the country specific publications: Bucsics et al., 2012; Bennie et al., 2013; Godman et al., 2013a; Hesse et al., 2013; Simoens et al., 2013).

**Figure 2:**
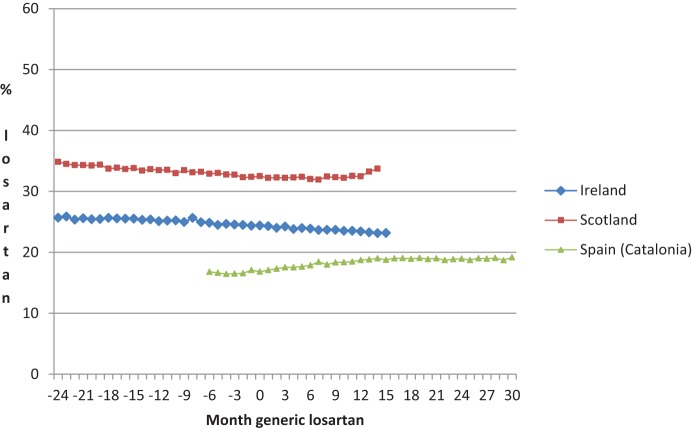
**Percentage utilization losartan vs. all single ARBs (DDD based) among the European countries with no multiple demand-side measures to preferentially enhance the prescribing of losartan (Table 1) before and after the availability of generic losartan (Time 0) on a monthly basis**.

Some countries instigated few or no specific demand-side measures to stimulate the preferential prescribing of losartan following generic availability. These were Ireland, Scotland, and Spain (Catalonia) (Table 1). This resulted in no change or even a fall in losartan use in Ireland as well as initially in Scotland (Figure 2). There was appreciable consistency between these three countries (Table 2), i.e., excluding the European countries that had instigated multiple demand-side measures from the analysis (Denmark, Sweden, Belgium, and Austria) resulted in very limited change in the post-generic utilization slopes with an average of 0.10% and an *SD* of 0.08% (Table 2).

Losartan utilization increased significantly in NHS Bury following the instigation of multiple demand-side measures, which were similar to Sweden (Table 1). These principally centered on the therapeutic switching of patients with hypertension from single-sourced ARBs to losartan (Martin et al., 2014). These multiple demand-side measures were introduced seven months after the availability of generic losartan. Prior to that, there was no change in the utilization of losartan post generic availability (Time Zero—Table 3) with no specific demand-side measures encouraging the preferential prescribing of losartan vs. single-sourced ARBs. Following the multiple demand-side measures (DM0) introduced in month eight after generic losartan, the utilization of losartan significantly increased from 26% of all single ARB items dispensed to 65% by the end of the study period (Table 3).

**Table 3:** **Utilization of losartan and other ARBs in NHS Bury (items dispensed) before and after generic losartan (month 0) and before and after multiple demand-side measures (MDM)**.

	**Months before generic losartan**					**Months after generic losartan**					**Months after multiple demand-side measures (MDMs)**							
											**MDM0**	**MDM1**	**MDM2**	**MDM3**	**MDM4**	**MDM5**	**MDM6**	**MDM7**
Month	−8	−6	−4	−2	0	1	3	4	6	7	8	9	11	12	13	14	15	16
Losartan	1169	1186	1328	1167	1288	1188	1229	1228	1200	1181	1399	1307	1552	1894	2171	2805	3187	3201
All orther ARBs	3739	3703	4077	3738	4021	3634	3610	3799	3613	3376	3873	3403	3654	3556	2853	2430	2234	1752
% losartan	24	24	25	24	24	25	25	24	25	26	27	28	30	35	43	54	59	65

Generic losartan accounted for up to 97–99% of total losartan (DDD basis) by the end of the study in Sweden and Scotland respectively. This was due to compulsory generic substitution in Sweden (Godman et al., 2009, 2013a) and high voluntary INN prescribing in Scotland (Bennie et al., 2013; Godman et al., 2013c). High INN prescribing rates were already seen in Scotland before generic losartan became available. There was lower utilization of generic losartan as a percentage of total losartan utilization (DDD basis) in Spain, Austria and Ireland. This was 80% in Spain (26% after 1 year), 46% in Austria and 24% in Ireland by the end of the study in each country.

The price of generic losartan (expenditure/ DDD) also varied considerably among the different European countries and regions. In Sweden, Denmark (total losartan), Scotland, and Austria, the price of generic losartan was 10, 12, 12, and 17% respectively of pre-patent loss prices by the end of the study. Prices for generic losartan were higher in Spain (Catalonia), Belgium (total losartan including both generic and originator losartan) and Ireland at 32, 54, and 56% respectively of pre-patent loss prices by the end of the study.

The combination of supply- and demand-side measures in Denmark resulted in a 77% reduction in overall ARB expenditure by the end of the study despite a 16% increase in utilization. Drug acquisition cost savings were estimated at over 290 million Danish Kroner (€40 million) per annum. In Sweden, total expenditure on single ARBs fell by 26% by the end of the study despite a 16% increase in utilization and in Belgium ARB expenditure fell by 15% by the end of the study despite a 1% increase in utilization. NHS Bury realized annual net savings of GB£290,000 (€348,000) for a population of 186,000 following the instigation of its multiple demand-side measures. This was eight times the cost of implementing their multifaceted approach. In Scotland, drug acquisition cost savings from low cost generics were estimated at GB£8 million (€9.6 million) per annum from the prescribing of generic vs. single-sourced losartan. These savings are growing in Scotland as more ARBs lose their patents.

## Discussion

Generic losartan created an opportunity for health authorities across Europe to realize considerable savings without compromising care. This is because separate studies, including therapeutic switching programmes, have shown that patient outcomes should not be compromised with such measures (Usher-Smith et al., 2008; Moon et al., 2010; Martin et al., 2014). A number of similar opportunities are pending with other patent expiries to provide future opportunities to health authorities (Jack, 2008; Godman et al., 2013d). The approach of some countries, e.g., Denmark, shows that these savings can be fully realized for pertinent classes. Aggressive changes in the reimbursement status of the single-sourced ARBs resulted in the prescribing of generic ARBs, i.e., losartan, in over 90% of cases. Without specific demand-side measures preferentially encouraging the prescribing of losartan, its prescribing remained stable or actually fell following generics (Figures 1, 2, Table 2).

Replicating the activities in Denmark throughout Europe would result in considerable savings with generic losartan priced at one 10th of the price of single-sourced products as seen in Scotland (Bennie et al., 2013). In other circumstances, such differences in the use of taxpayers' monies would result in serious public debate. Here, it is more likely that the influence of pharmaceutical companies promoting their single-sourced ARBs, the potential dissociation of the prescriber from the budget holder, other health authority priorities or a combination of these, resulted in the appreciable differences in physician prescribing behavior post generic losartan (Figure 1). It is perhaps sufficient to say that if all European countries mirrored the activities in Denmark (Table 1), billions of euros could have been redirected without compromising care with all ARBs seen as therapeutically similar at appropriate doses. Multiple demand-side measures in NHS Bury and Sweden, including therapeutic switching, also produced considerable savings even when factoring in the cost of implementation including physician time from switching patients from single-sourced ARBs to losartan (Godman et al., 2013a; Martin et al., 2014). These two countries and regions provide examples where it is difficult for health authorities to delist single-sourced products in a class from the reimbursement list.

Some interventions were less effective than others. Lifting prescribing restrictions for losartan but not the other ARBs in Austria and Belgium (Table 1) significantly enhanced its use (Figure 1) (Bucsics et al., 2012; Simoens et al., 2013). However, the resultant change in its utilization was lower than the changes seen in either Denmark, Sweden, or NHS Bury post generic losartan (Figure 1, Table 3). Having said this, Figures 1, 2 and Tables 1, 2 demonstrate again that multiple demand-side measures are needed to change physician prescribing habits (Austria, Belgium, Denmark and Sweden vs. Ireland, Scotland, and Spain [Catalonia]). This has previously been seen with the proton pump inhibitors and statins following the availability of generic omeprazole and simvastatin respectively (Godman et al., 2010a,b), as well as with demand-side measures to enhance the prescribing of generic ACEIs vs. single-sourced ARBs (Voncina et al., 2011; Godman et al., 2013c). This has also been seen in other situations (Bero et al., 1998; Barton, 2001).

The findings in Scotland and initially NHS Bury also suggest there is no “spill over” learning from one class to another to effect changes in physician prescribing habits even if the classes are closely related. Multiple demand-side measures (education, engineering', and economics) had appreciably limited the utilization of single-sourced ARBs vs. generic ACEIs in Scotland compared with countries with few demand-side measures (Voncina et al., 2011; Godman et al., 2013c). However, these learnings were not carried through into the preferential prescribing of losartan vs. single-sourced ARBs once it became available (Bennie et al., 2013; Martin et al., 2014). This changed in NHS Bury once multiple demand-side measures were introduced to significantly enhance the prescribing of losartan vs. single-sourced ARBs (Martin et al., 2014). One mitigating reason for the lack of a “spill over” effect in Scotland, and initially in NHS Bury, could be that the complexity of the message is enhanced, i.e., health authorities going from advocating ACEIs first line vs. ARBs to advocating ACEIs and low cost ARB first line vs. single-sourced ARBs (Bennie et al., 2013).

There appeared to be no problems with generic losartan in clinical practice as it accounted for up to 97–99% of total losartan (DDD basis) by the end of the study period in Sweden and Scotland respectively (Bennie et al., 2013; Godman et al., 2013a). However, we cannot say this with certainty as we did not have access to patient data. The high voluntary INN prescribing rates with losartan in Scotland mirror those seen with other products and classes (Godman et al., 2013c). This starts with educating students in medical school (Voncina et al., 2011; Godman et al., 2013c), and provides guidance to countries where the dispensing of different branded generics without adequate explanations can cause confusion if patients do not receive adequate information about their medicines (Godman et al., 2009; Olsson and Kalvemark Sporrong, 2012). This can lead to either duplication of medicines; alternatively, patients not taking their prescribed treatments as directed. Consequently, not gaining the most benefit from the medicines prescribed (Olsson et al., 2014). These scenarios are exacerbated if pharmacists lack training on how to handle concerns with substitution and/ or do not receive adequate payment for providing relevant information to patients potentially limiting their time with them (Olsson and Kalvemark Sporrong, 2012; Martin et al., 2014). INN prescribing, apart from a limited number of well-known situations, is one way to address this, which has worked well in the UK (McGinn et al., 2010; Godman et al., 2013c).

There were also considerable differences in prices of generic losartan among the different countries and regions. This reflects differences in their policies to enhance the utilization of generics as well as the different pricing policies for generics. The low prices for generic losartan in Scotland, which is similar to those for other generics, follows reforms in the UK to enhance transparency in the cost of producing generics as well as the discounts offered by manufacturers to wholesalers and pharmacists to preferentially dispense their generic (Voncina et al., 2011; Bennie et al., 2012, 2013; Godman et al., 2013c). The price reduction in Sweden, which is also similar to other generics, is a result of introducing compulsory generic substitution with the lowest priced molecule (Andersson et al., 2005; Godman et al., 2009, 2013a). Generic prices are falling further in Sweden with the recent introduction of monthly auctions, with the manufacturer winning the auction guaranteed a considerable proportion of dispensed generics the following month (Godman et al., 2012a,b). The modest price reduction for generic losartan in Belgium reflects the current situation where generic companies only have to lower their prices to the reference price level to be reimbursed. This was only 16% vs. pre-patent loss prices until 2002, 20% until 2003, 26% until 2005, and currently 31% (Godman et al., 2013e; Simoens et al., 2013). The relatively high price for generic losartan in Ireland reflects limited measures to date to reduce generic prices, although this is now changing (Godman et al., 2010a; Cahir et al., 2012). Consequently, measures to increase the attractiveness of the generic market, as well as enhance the transparency in their pricing as seen in Sweden and the UK, provide guidance to countries seeking ways to achieve further savings from the availability of generics.

We appreciate that we have not measured patient outcomes in any of the European countries and regions following the various initiatives apart from NHS Bury. However as stated, surveillance and other studies have shown no compromise on patient outcomes following measures to increase the prescribing of low cost ARBs (Usher-Smith et al., 2008; Moon et al., 2010; Svanstrom et al., 2012; Martin et al., 2014).

## Conclusion

The loss of market exclusivity for medicines can create considerable opportunities for European health authorities to save resources to help fund increased drug volumes and new premium priced drugs (Godman et al., 2014). This losartan case study shows that multiple demand-side measures can be extremely effective with influencing subsequent physician prescribing. Without these, prescribing rates for multiple sourced products in a class can actually fall. However, some interventions are less effective than others. If all European countries followed the example of Denmark, NHS Bury in later years, or Sweden, considerable resources could have been saved. Having said this, there does appear at times to be a disconnect between the physician prescriber and the payer of medicines, creating a dissociation of responsibility which is reducing cost-effective prescribing. If we are to maintain the European ideals of comprehensive and equitable healthcare, we must urgently address this.

## Funding

The analysis and report writing was in part funded by a grant from the Karolinska Institutet.

## Author contributions

Details of the contributions are as follows: James C. Moon, Brian Godman, and Rickard E. Malmström devised the concept for the paper and produced the first and subsequent drafts. Max Petzold performed the statistical analyses and critiqued successive drafts. Samantha Alvarez-Madrazo, Kathleen Bennett, Iain Bishop, Anna Bucsics, Ulrik Hesse, Andrew Martin, Steven Simoens, Corinne Zara, and Rickard E. Malmström provided the utilization and expenditure data for their respective countries as well as details on the demand-side measures. They also critiqued successive drafts.

### Conflict of interest statement

Some co-authors are employed by health authorities or health insurance companies. These include: Iain Bishop (Scotland), Anna Bucsics (Austria), Ulrik Hesse (Denmark), Corinne Zara (Barcelona, Spain), and Rickard E. Malmström (Stockholm, Sweden). Steven Simoens holds the European Generic Medicines Association (EGA) Chair “European policy toward generic medicines.” Otherwise the authors declare that the research was conducted in the absence of any commercial or financial relationships that could be construed as a potential conflict of interest.
